# Antiviral activity of mitoxantrone dihydrochloride against human herpes simplex virus mediated by suppression of the viral immediate early genes

**DOI:** 10.1186/s12866-019-1639-8

**Published:** 2019-12-07

**Authors:** Qiang Huang, Jue Hou, Peng Yang, Jun Yan, Xiaoliang Yu, Ying Zhuo, Sudan He, Feng Xu

**Affiliations:** 1 Department of Obstetrics and Gynecology, Suzhou Dushuhu Public Hospital (Soochow University Multi-Disciplinary Polyclinic), Suzhou, China; 2 Blood Research Laboratory, Chengdu Blood Center, Chengdu, Sichuan 610041 China; 3 Cyrus Tang Hematology Center, Collaborative Innovation Center of Hematology, Jiangsu Institute of Hematology, the First Affiliated Hospital, Jiangsu Key Laboratory of Preventive and Translational Medicine for Geriatric Diseases, Soochow University, Suzhou, China; 4 Department of emergency medicine, First Affiliated Hospital, Soochow University, 1 Shizi Rd, Suzhou, China; 5 Department of Pulmonology, First Affiliated Hospital, Soochow University, Suzhou, China

**Keywords:** Herpes simplex virus, Anti-HSV drugs, Mitoxantrone dihydrochloride

## Abstract

**Background:**

HSV-1 is a common pathogen that infects 50–90% of the human population worldwide. HSV-1 causes numerous infection-related diseases, some of which are severely life-threatening. There are antiviral medications with activity against HSV-1. However, with the emergence of drug-resistant mutant strains of HSV-1, there is an urgent need to develop new effective anti-HSV-1 agents.

**Methods:**

Therefore, we screened a chemical library of approximately 1500 compounds to identify inhibitors of HSV-1-induced toxicity for further drug development. Moreover, we performed several experiments, including western blot analysis, Q-PCR analysis and luciferase activity assay, to explore the antiviral mechanism of the candidates.

**Results:**

Here, we identified a small molecule, mitoxantrone dihydrochloride, with potency against HSV-1-induced toxicity. Furthermore, the viral titers and expression levels of HSV-1 viral proteins were potently reduced by the presence of MD in many cell lines. Using Q-PCR analysis, we found that MD efficiently reduced the transcription of viral genes that are essential for DNA synthesis, namely, *UL5, UL9, UL29, UL30, UL42* and *UL52*. Notably, MD also significantly inhibited the transcription of the immediate early genes *ICP0, ICP22, ICP27* and *ICP47*, all of which are required for the expression of early and late viral gene products. Using immunofluorescence and western blot analysis, we found that the antiviral effect of MD was independent of the activation of the NF-κB and MAPK pathways. Furthermore, we found that the reduction in the transcription of viral immediate early genes was not related to the promoter activities of *ICP0*.

**Conclusions:**

Therefore, the identification of compound MD as an inhibitor of toxicity induced by HSV-1 highlights its potential use in the development of novel anti-HSV-1 drugs.

## Background

Herpes simplex virus-1 (henceforth HSV-1) is a double-stranded DNA pathogen that possesses a large genome of approximately 150 k nucleotides [1]. HSV-1 is recognized as one of the most common human pathogens infecting 50–90% of the population [2]. After infection, HSV-1 is dormant in sensory neurons and persists for the lifetime of the host [3, 4]. When the immune state of the host is compromised, the virus can be reactivated by the proper stimulus. Although HSV-1 mainly causes innocuous diseases, such as labialis, pharyngitis and keratitis, it can also result in serious life-threatening diseases, including encephalitis [5–9].

Mature HSV-1 contains a large DNA genome core enveloped by a protein capsid. The middle layer is referred to as tegument, which is composed of viral proteins and mRNA. The outer layer is surrounded by the glycoproteins gB, gC and gD, all of which are necessary for viral entry [10, 11]. Once the HSV-1 virus enters the cytoplasm of the infected cell, its genome is released into the cell nucleus and encodes several gene products that enable viral replication [12, 13]. Viral gene expression is strictly regulated in a cascade fashion. Immediate early genes, early genes and late genes are expressed successively [14, 15]. The immediate early genes include *ICP0, ICP4, ICP22, ICP27* and *ICP47*, whose products are required to initiate and regulate the efficient expression of early genes and late genes [16, 17]. For example, ICP0 mediates the degradation of several cellular proteins and induces the conjugation of ubiquitin. Mutation of *ICP4* or *ICP27* significantly blocked the expression of early and late viral genes [18]. Regulated by the immediate early genes, early genes are expressed approximately 2–8 h after infection. Most early genes are involved in viral replication. As the infection progresses the late genes begin to be expressed, and the products of late genes are structural proteins whose expression also depends on immediate early genes. Some HSV-1 viral proteins are known to be necessary for viral DNA synthesis and include proteins encoded by the *UL5, UL8, UL29, UL30, UL42* and *UL52* genes [19–21]. For example, UL9 helps to unwind the DNA strain by flanking the origins of DNA replication. UL30 and UL42 are two subunits of the DNA polymerase, so losing one of these proteins impedes the elongation of viral DNA chains.

There is a number of available antiviral medications with activity against HSV-1. As a nucleoside analogue, acyclovir is commonly used for the treatment of HSV-1 infection. Because its structure is similar to that of a nucleoside, acyclovir serves as a substrate for the viral DNA polymerase and terminates the extension of the viral DNA to inhibit the proliferation of the HSV-1 virus [22, 23]. Peciclovir and foscarnet can also hinder the elongation of HSV DNA by similar mechanisms of action as acyclovir [24]. However, with the increasing drug resistance of HSV-1 to acyclovir, research and development of new effective anti-HSV-1 agents is becoming increasingly important. It has been reported that the small molecular compound mitoxantrone dihydrochloride (henceforth MD) is a topoisomerase II inhibitor that shows promising efficacy in clinical trials targeting lymphomas, breast cancer, and prostate cancer [25–27]. In the current study, using a compound library screen, we found that MD effectively blocked not only HSV-1-induced toxicity but also viral titers in multiple cell lines. MD also showed a potent effect on the expression of essential HSV-1 genes without activating the NF-κB and MAPK pathways or the ICP0 promotor.

## Results

### Screening of small molecular inhibitors against HSV-1

**Fig. 1:**
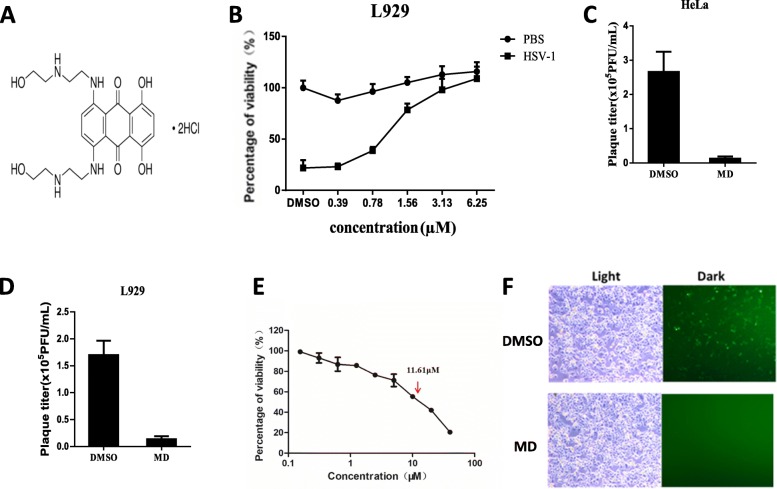
MD inhibited HSV-1 induced toxicity and viral proliferation in both human and mouse cells. **a** The structure of the Mitoxantrone dihydrochloride. **b** L929 cells were pretreated with DMSO or the indicated concentrations of MD for 1 h, then the cells were infected with HSV-1(MOI = 2) for 18 h. Identical MOI was used in later experiments unless otherwise stated. The cell-survival rate was determined by measuring ATP levels. **c** HeLa cells and (**d**) L929 cells were pretreated with DMSO or MD (3.0 μM) for 1 h, then the cells were infected with HSV-1 for 6 h. Viral titers were determined by plaque forming assay. **e** L929 cells were pretreated with the indicated concentrations of MD for 18 h, then the cell-survival rate was determined by measuring ATP levels. **f** HeLa cells were pretreated with DMSO or MD (3.0 μM) for 1 h, then the cells were infected with green fluorescent protein (GFP) labeled HSV-1 for additional 16 h. Images were captured by inverted fluorescence microscope

In our previous study, we screened the LOPAC small-scale library to identify small molecules that can inhibit HSV-1-induced toxicity [28] and identified MD as a potential inhibitor of human herpes simples virus. MD has been reported as a topoisomerase II inhibitor. It has been used as an approved chemotherapy drug to treat lymphoma and prostate cancer in consideration of its antitumour activity. Since its effect on HSV-1 was unknown, we further tested the antiviral effect of MD on HSV-1. L929 cells were treated with various concentrations of MD for 1 h prior to HSV-1 infection. As shown in Fig. 1b, we found that HSV-1-induced cell death was inhibited significantly in a dose-dependent manner with an estimated IC:50 value of 1.21 μM. MD treatment with an estimated CC:50 value of 11.6 μM completely suppressed HSV-1-induced toxicity at 3.13 μM and had no obvious cytotoxicity to cells under this concentration (Fig. 1b and e). We further estimated whether the HSV-1 titers were affected by the present of MD by plaque forming assay. The results showed that the viral titers were significantly suppressed by MD in HeLa cells (t = 7.56, *P* < 0.005) and L929 cells (t = 10.02, *P* < 0.005) (Fig. 1c and d). Moreover, the HeLa cells were infected with GFP-labelled HSV-1 to observe the efficacy more directly. We found that the GFP-labelled HSV-1 virus was significantly inhibited by MD compared with the DMSO control (Fig. 1f). Thus, these data show that MD robustly inhibits HSV-1-induced toxicity and viral replication.

### MD reduces the expression levels of HSV-1 proteins in both human and mouse cells

**Fig. 2:**
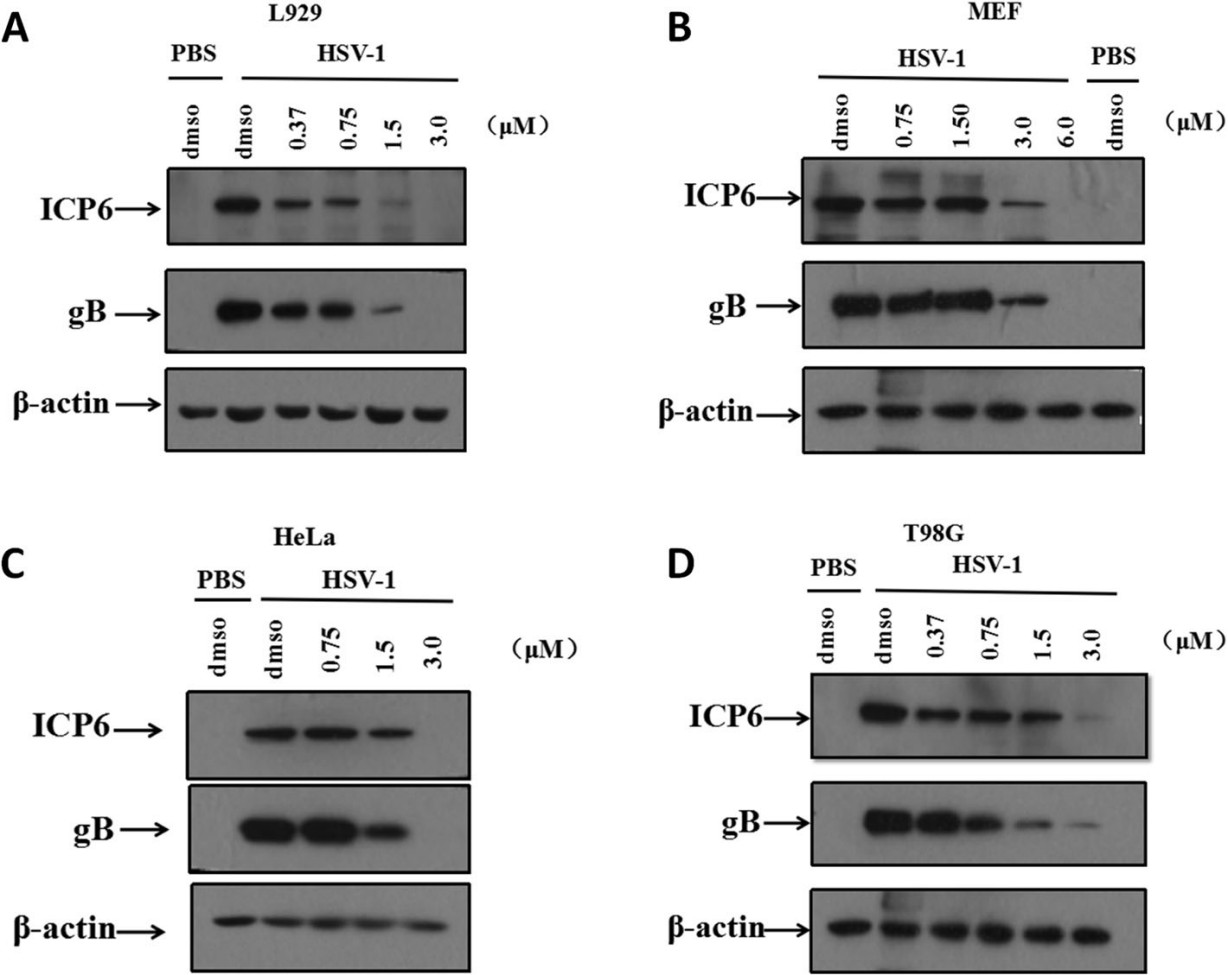
MD significantly suppressed the expression levels of HSV-1 proteins. L929 (**a**), MEF(**b**), HeLa(**c**) and T98G(**d**) were pretreated with DMSO or MD at indicated concentration for 1 h prior to infection with HSV-1 for additional 6 h. Viral proteins were harvested from cell lysates and the expression levels of gB and ICP6 were detected by western blot analysis

HSV-1 glycoproteins including gB, gD, gH and gL are required for HSV-1 entry and cell fusion [10, 11]. ICP6 is a HSV-1 ribonucleotide large subunit that is critical for the synthesis of deoxyribonucleotide [29]. We then investigated whether MD suppressed the expression levels of HSV-1 proteins. In this study, the results of western blot analysis showed that the expression levels of gB and ICP6 were significantly suppressed in a dose-dependent manner in both the mouse cell line (Fig. 2a and b) and the human cell line (Fig. 2c and d).

### MD shows antiviral efficacy even after HSV-1 entry

**Fig. 3:**
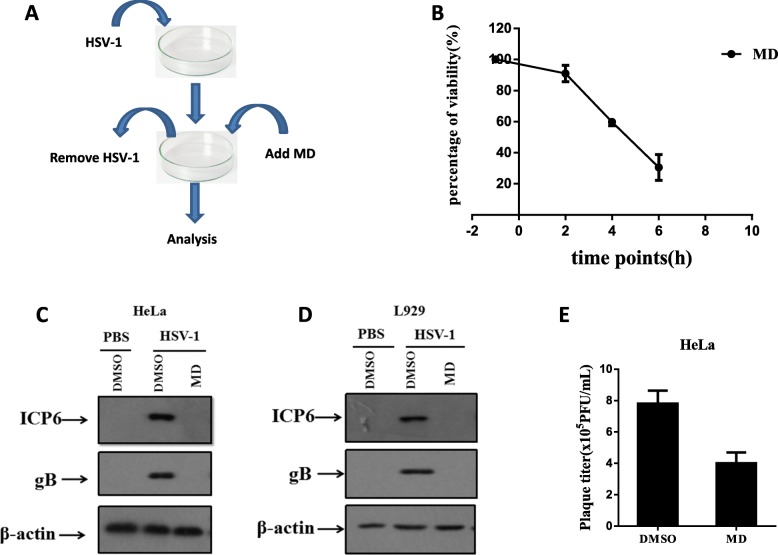
MD inhibited viral replication and the expression levels of HSV-1 proteins even after HSV-1 entry. **a** Schematic view. **b** L929 cells were infected with HSV-1 and washed twice with PBS at the indicated time points. Then cells were cultured with fresh medium added with MD (3.0 μM) for additional 18 h. Then the cell survival rate was determined by measuring ATP levels. HeLa (**c**) and L929 (**d**) was infected with HSV-1 for 2 h, then the cells were washed twice with PBS and cultured in viral free medium containing DMSO or MD (3.0 μM) for additional 6 h. The expression levels of viral proteins were measured by western blot analysis. The viral titers in HeLa cells (**e**) were measured by plaque forming assay

Viral infection is a complex procedure consisting of entry into host cells, replication and transcription of the viral genome, viral protein synthesis, viral assembly and release [10]. Antiviral medications acting at any step of these processes will block viral infection. To investigate whether MD inhibited the expression levels of viral proteins even after HSV-1 entry, we washed cells with PBS at the indicated time points after HSV-1 infection at which HSV-1 had completed entry (Fig. 3a). Then, the cells were cultured with fresh medium supplemented with MD for an additional 18 h. We found that MD inhibited HSV-1-induced toxicity even after HSV-1 cell entry. (Fig. 3b). However, the later the MD was added, the worse the antiviral efficacy. MD consistently inhibited the expression of viral proteins in both human and mouse cells even after HSV-1 entered cells (Fig. 3c and d). Viral titers were also reduced in HSV-1-infected cells by post-treatment with MD (t = 7.76, *P* < 0.005) (Fig. 3e). Overall, MD likely exhibits antiviral efficacy by suppressing the expression levels of HSV-1 proteins rather than blocking entry of virus into host cells.

### MD suppresses the transcription of viral genes

**Fig. 4:**
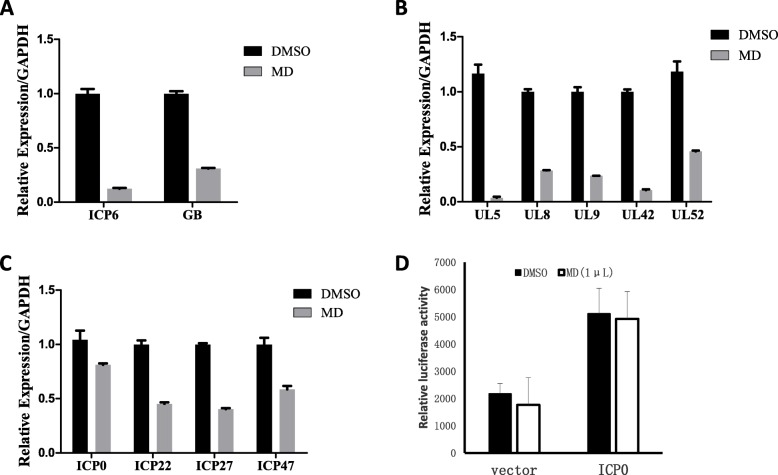
MD suppressed the transcription of viral genes. **a**-**c** L929 was pretreated with DMSO or MD (3.0 μM) for 1 h prior to HSV-1 infection for additional 2 h. The expression levels of indicated genes were measured by quantitative PCR. The 293 T cells were transfected with the vector and ICP0 promoter after treated with DMSO and MD (1 μM) for 1 h. Cells were harvested to determine luciferase activity 36 h post transfection (**d**)

Given that MD inhibited viral toxicity and suppressed the expression levels of viral proteins, we further confirmed whether the transcription levels of viral genes were also affected by MD. To examine the effects of MD on HSV-1 gene expression, total RNA was extracted from the treated cells, and Q-PCR was performed using specific primers as described in the Methods. The transcription levels of the *ICP6* and *GB* genes were reduced by MD (Fig. 4a). This result was consistent with the observation of the protein levels by western blot (Fig. 2). HSV-1 genes such as *UL5*, *UL8*, *UL9*, *UL42* and *UL52* are required for viral DNA replication. We found that the transcription levels of these genes were also inhibited by MD (Fig. 4b). Remarkably, MD also reduced the transcription levels of the immediate early genes *ICP0, ICP22, ICP27, and ICP47,* all of which are required for the expression of early and late viral gene products (Fig. 4c). These results suggest that MD inhibits HSV-1 replication by suppressing the expression of immediate early genes.

Having shown that MD suppressed the transcription of immediate early genes, we sought to characterize whether MD affects the promoter activity of immediate early genes. We generated a pGL4.17-based luciferase construct containing the ICP0 promoter sequence. Using a luciferase reporter assay, we found that MD had no effect on the promoter activity of *ICP0,* which suggests that MD interfered with the transcription of viral immediate early genes without affecting the promoter activity of *ICP0* (Fig. 4d).

### MD has no effect on NF-κB or MAPK activation

**Fig. 5:**
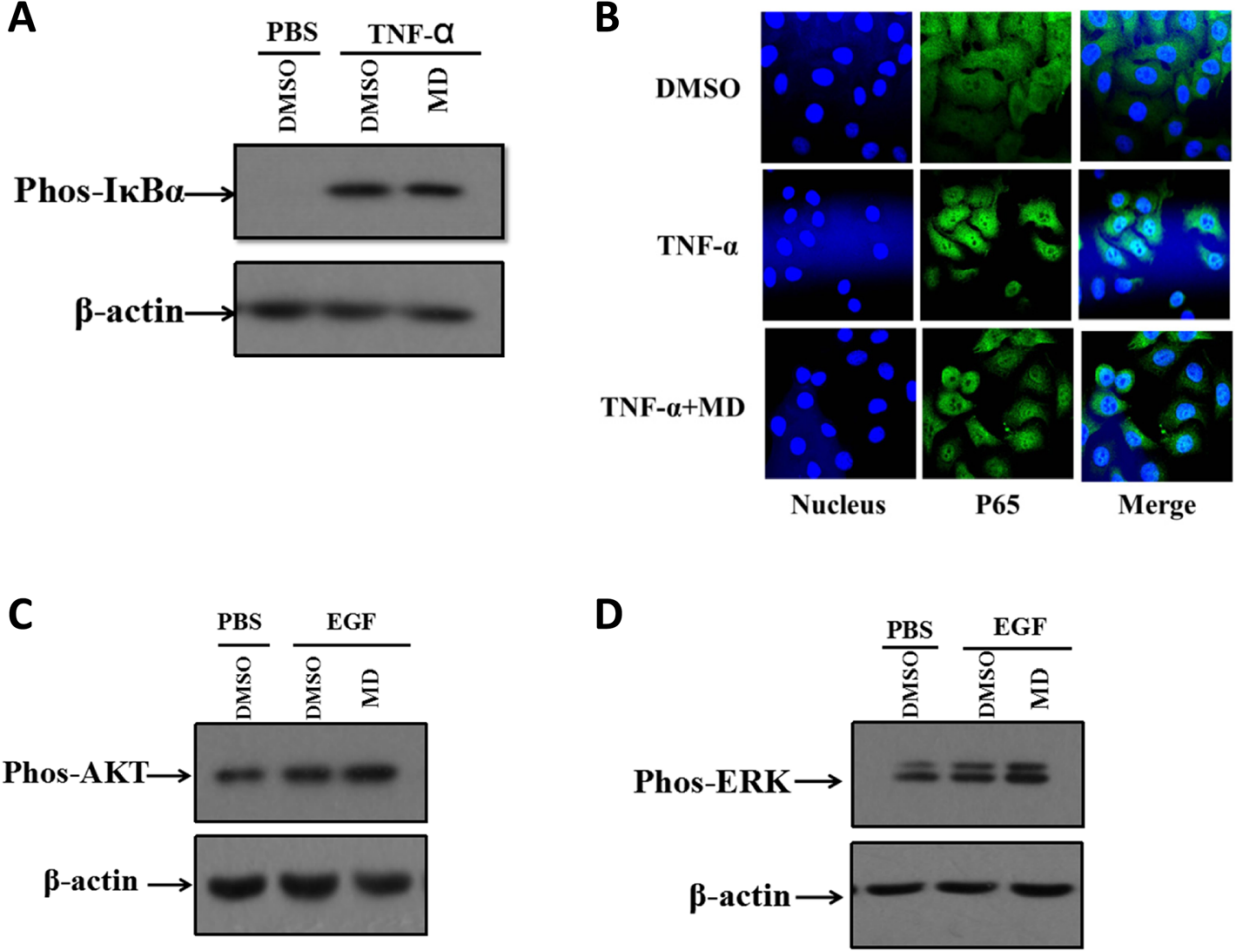
The activations of NF-κB and MAPK signaling pathways and ICP0 promotor activity were not affected by MD. **a** HeLa were pretreated with DMSO or MD (3.0 μM) for 1 h prior to stimulation with TNF-α (100 ng/mL) for 15 min. Then some of the HeLa cells were subjected to western blot to analyze the occurrence of IκBα phosphorylation. The rest of HeLa cells were stained with anti-P65 antibody (green) and DAPI (blue). The pictures were captured by confocal laser scanning microscope. **b** RD cells were cultured in serum free medium for 5 h. Then cells were treated with DMSO or MD (3.0 μM) and cultured in serum free medium for additional 1 h. EGF was added for 15 min to stimulate activation of MAPK pathway. The occurrence of AKT (**c**) and ERK (**d**) phosphorylation were analyzed by western blot analysis

We proceeded to explore the molecular mechanism by which immediate early genes were inhibited by MD. It has been reported that efficient replication of HSV-1 relies on the activation of the NF-κB pathway in the host cell [30–32]. We further tested whether MD suppressed the expression of viral genes by disturbing the NF-κB pathway. Both viral evasion and antiviral cytokines such as TNF-α are able to induce activation of the NF-κB pathway, so we used recombinant protein TNF-α to trigger the pathway. As shown in Fig. 5a, MD did not abolish TNF-α-induced phosphorylation of IκBα and P65 (Fig. 5a). Using confocal microscopy, we found that MD did not block nuclear translocation of the P65 protein in response to TNF-α stimulation, which was consistent with the western blot results (Fig. 5b). Therefore, MD had no effect on the NF-κB signalling pathway. Moreover, the MAPK signalling pathway has also been reported to be involved in the regulation of cytokine production and viral replication [32–34], so we also investigated whether this pathway was affected by MD. We found that MD showed no effect on the activation of ERK and AKT, which are critical components of the MAPK pathway (Fig. 5c, d). Thus, all these data show that MD exhibits anti-HSV-1 activity independent of the cellular NF-κB and MAPK pathways.

## Discussion

HSV-1 is a highly contagious virus that establishes a lifelong latent infection in host cells. During the latent period, the virus infects sensory neurons near the site of primary infection, and the virions hide in the nucleus of the neuron. When the virus is reactivated by the proper stimulus, the virus initiates a lytic infection. HSV-1 infects the epithelium and initiates lytic replication, which further leads to cellular toxicity and tissue lesions. With the participation of cellular transcription factors, HSV-1 genes are transcribed by cellular RNA polymerase II in a highly regulated cascade. Once the viral genome invades the host nucleus, the immediate early genes, including ICP0 and ICP4, play an essential role in initiating viral transcription. Regulated by the immediate early genes, early genes are expressed approximately 2–8 h after infection. The products of early genes are involved in viral replication, while the products of late genes are usually structural proteins [14, 15].

HSV-1 is a threat to human health and causes a number of diseases ranging from simple cold sores to keratitis and lethal encephalitis [5–9]. Current anti-HSV-1 drugs including acyclovir, penciclovir and foscarnet are viral DNA synthesis inhibitors. Acyclovir and penciclovir are commonly used for the treatment of HSV-1 infection. Phosphorylated by virus-encoded thymidine kinases, acyclovir serves as a substrate for the virus DNA polymerase and prevents chain elongation and synthesis of the viral DNA. Penciclovir exerts antiviral activity by a similar mechanism as acyclovir [22, 23]. Foscarnet is a phosphonic acid derivative that inhibits HSV-1 replication by inhibiting the pyrophosphate site on the herpesvirus DNA polymerase [24]. However, with the chronic administration of anti-HSV drugs, drug-resistant HSV-1 strains have become a major threat worldwide. In particular, patients with immunodeficiencies are prone to develop drug-resistant HSV-1 strains. Considering that attempts to develop a vaccine against HSV-1 have been found to be ineffective, there is an urgent need to develop new effective antiviral drugs.

It has been reported that MD has antitumour effects, especially against lymphoma, breast cancer and prostate cancer, by targeting DNA topoisomerase II [25–27]. However, its anti-HSV-1 effect has not been reported. In our study, we found that MD suppressed HSV-1-induced toxicity at 3.13 μM and had no obvious cytotoxicity to cells at this concentration. Further experiments demonstrated that MD suppressed GFP-tagged HSV-1 replication and viral titers in host cells. We tested our hypothesis, that MD shows antiviral activity against HSV-1 by inhibiting the expression levels of viral proteins. We found that the expression levels of viral genes, including ICP6 and gB, were significantly suppressed by western blot analysis. Therefore, we found that MD blocked HSV-1-induced toxicity and suppressed viral protein synthesis. Given that MD can interact with topoisomerase II to regulate DNA synthesis, examination of whether MD reduces the expression level of viral genes by directly binding topoisomerase II is a promising direction to evaluate the possible mechanism of antiviral efficacy of MD in the future.

Viral infection is a complex process consisting of virus entry into host cells, replication and transcription of the viral genome, viral protein synthesis, viral assembly, and release [35]. Antiviral medications act by impeding any of these steps. To elucidate whether MD protects cells from HSV-1 infection at viral entry or post viral entry, we changed the order in which HSV-1 and MD were added to cells. The results showed that MD still suppressed viral protein synthesis and viral titers in host cells even after the virus had entered the host cells. Therefore, MD showed antiviral efficacy mainly by suppressing viral protein synthesis rather than blocking the entry of viruses into host cells.

The transcription factor NF-κB, a heterodimer of the P65 and P50 subunits, is involved in many cellular events, such as innate and adaptive immunity and inflammation. The efficient replication of HSV-1 relies on the activation of the NF-κB pathway in host cells. Moreover, it has been reported that the MAPK signalling pathway is also involved in the regulation of cytokine production and viral replication [32]. Therefore, we focused on whether MD inhibits HSV-1 replication by blocking the NF-κB and MAPK pathways. According to our results, the phosphorylation of IκBα and the nuclear translocation of the P65 protein were affected by MD treatment. In addition, we did not find that MD had any effect on the activation of the MAPK pathway. Therefore, MD exerts antiviral activity in a manner that is dependent on disturbing the activation of the NF-κB and MAPK pathways.

MD interrupted viral protein synthesis, so we also examined whether the transcription of viral genes was interrupted in the presence of MD. We found that the transcription of *UL5, UL9, UL29, UL30, UL42* and *UL52*, all of which are necessary for viral DNA synthesis, was inhibited by MD. Moreover, the HSV-1 immediate early genes play important roles in regulating the expression of the early genes and late genes. Notably, we also found that the transcription of immediate early genes *ICP0, ICP22, ICP27, and ICP47,* was also reduced in the presence of MD. Therefore, MD inhibits viral protein synthesis by interrupting the transcription of viral immediate early genes.

As one of the immediate early proteins, ICP0 has many biological functions. Some studies found that HSV-1 ICP0 mutant viruses might yield avirulent progeny, suggesting that ICP0 plays a pivotal role during lytic and latent infection [36, 37]. However, according to our results, MD had no effect on the promoter activity of *ICP0,* which suggests that MD interrupts the transcription of viral immediate early genes without affecting the promoter activity of *ICP0*. It is still an interesting future direction to examine the promoter activities of other immediate early genes to explore the precise molecular mechanism of action of MD against HSV-1.

## Conclusions

In conclusion, mitoxantrone dihydrochloride is an interesting candidate for the development of a novel therapeutic drug against HSV-1 infectious disease that relies on its effective anti-HSV-1 activity.

## Methods

### Antibodies and reagents

The following antibodies were used for Western-blotting: β-actin (A2066; Sigma), gB (6506; Abcam), p-ERK (4370; cell signaling) and p-IκBα (9246; Cell Signaling). Mitoxantrone dihydrochloride was purchased from sigma (M6545). HSV-1 ICP6 polyclonal antibody was generated against the N-terminal peptide of ICP6. TNF-α recombinant protein was generated according to previously instruction [38].

### Cells and viruses

Mouse fibroblast cells (L929) and African green monkey kidney cells (Vero) in which the viruses were propagated was from ATCC (American Type Culture Collection). Mouse embryonic fibroblasts (MEFs) were isolated from day 14.5–15.5 embryos. HSV-1 KOS strain was kindly provided by Dr. Sandra K. Weller (University of Conecticut Health Center) and GFP-labeled HSV-1 F stain was kindly provided by Dr. Chunfu Zheng (Soochow University).

### Cell survival

Cell Titer-Glo Luminescent Cell Viability Assay kit (Promega) was used to determine cell survival by measuring ATP levels according to the manufacturer’s instruction.

### Plaque forming assay

The infection of cells was performed at an MOI of 2 for 6 h. The harvested cells were frozen and thawed three times and then spun down at 13000 x g for 5 min. The supernatant containing virus was collected to infect cells for 12 h. The cells then were stained with crystal violet solution (2%) Then by using the Inverted microscope, the viral plaque formation were counted.

### Western blot analysis

Cell pellet was collected by centrifugation at 13000 x g for 1 min. The harvested cells were washed once with PBS and then resuspended in lysis buffer consist of 20 mM Tris–HCl, PH 7.4, 150 mM NaCl, 10% glycerol, 1% Triton X-100, 1 mM Na3VO4, 25mMβ-glycerolphosphate, 0.1 mM PMSF, a complete protease inhibitor set (Roche). The resuspended cell pellet was vortexed for 20s. lysis on ice for 20 min before cell lysates were centrifuged at 13000 x g for 20 min at 4 °C. The supernatants which the soluble proteins are in were collected and subjected to western blot analysis. The proteins were detected by using appropriate antibody.

### Determination of viral RNA levels

Viral total RNA for real-time PCR was extracted by using TRIzol isolation (Invitrogen). Reverse transcription reactions were performed with reverse transcriptase (Life technology). Levels of various genes were finally analyzed by real-time PCR. The real-time was performed using specific primers for the ICP6, ICP0, ICP22, ICP27, ICP47, UL5, UL8, UL9, UL42, UL52. The sequence of primers used for Q-PCR analysis is shown in Additional file 1: Table S1.

### Cell infected with GFP-labeled virus imaging

HeLa cells were seeded into the 6-well plates at a density of 3 × 10^6^ and were infected with GFP-labeled HSV-1(MOI = 2) for 8 h. Cells were analyzed for GFP by Leica DMILLED inverted microscope.

### Transfection and luciferase reporter assay

The 293 T were pretreated with DMSO and MD (1 μM) for 1 h. Then the 293 T cells were co-transfected with pGL4.17 containing the ICP0 promotor sequence and RL-TK plasmids by using lipofectamine 2000 reagent (Invitrogen). Cells were harvested to determine luciferase activity 36 h post transfection using the Dual-Luciferase Reporter Assay System kit (Promega, WI, USA, E1980) by a luminometer instrument.

### Immunofluorescence technique

HeLa cells were seeded on glass coverslips at a density of 3 × 10^4^cells/plate and cultured overnight. The cells were pretreated with DMSO or MD for 1 h. Then, the cells were washed twice with PBS after treatment with TNF-α (100 ng/mL) for about 15 min. Fix the cultured cells for 15 min with 4% paraformalde-hyde and Permeate the cells at room temperature for 15 min with 0.5%Triton X-100. Cells were blocked with 5% bovine serum albumin (BSA) in PBS for 30 min, then the primary antibody and secondary antibody was added successively. Nuclear was stained with DAPI. Image were observed and collected under a confocal laser scanning microscope.

### Statistical analyses

Data of cell-survival rate are represented as the mean ± SD of duplicates. All experiments were repeated at least three times with similar results. Significance was evaluated using t tests (GraphPad Prism software).

## Additional file

**Additional file 1.** List of PCR primers used in Q-PCR assay.

## Data Availability

All data generated or analyzed during this study are included in this published article.

## Abbreviations

HSV-1: Herpes simplex virus-1
MD: Mitoxantrone dihydrochloride
NF-κB: Nuclear Factor-κB
TNF-α: Tumor necrosis factor-α
