# Supplementary material for: Antiviral activity of mitoxantrone dihydrochloride against human herpes simplex virus mediated by suppression of the viral immediate early genes
Source: BMC Microbiol. 2019 Dec 7;19:274. doi: 10.1186/s12866-019-1639-8 (PMC6898960; doi:10.1186/s12866-019-1639-8)
Supplement: Supplementary file 1 — Additional file 1. List of PCR primers used in Q-PCR assay. [file 12866_2019_1639_MOESM1_ESM.doc]

**List of PCR primers used in Q-PCR assay**

ICP6-F GAGCCCCTTCGTCATGTTCA

ICP6-R AGTCAAACGTCTGCCTGGAG

GB-F GGACATCAAGGCGGAGAACA

GB-R TTCTCCTTGAAGACCACCGC

ICP0-F CCTGTCGCCTTACGTGAACA

ICP0-R CCATGTTTCCCGTCTGGTCC

ICP22-F GAAATCTCCGATGCCACCGA

ICP22-R TCTGGGGTTTCCAGCGTAAC

ICP27-F CCGAGCCTCTATCGCACTTT

ICP27-R GTCCCGATAATGGGGTCCTG

ICP47-F TACCGGATTACGGGGACTGT

ICP47-R ATAAAAGGGGGCGTGAGGAC

UL5-F GATGACGATCACGTTGCTGC

UL5-R CCCTCAGGGAGTTTCCGTTC

UL8-F ATTTTAGTGGCGGGATGCCA

UL8-R CCGTTAACATCACCACCGGA

UL9-F GCAGCAGGCGTAGCATTAAC

UL9-R GGGTTCACCCGAAAACAACG

UL42-F TGTTCACCACGAGTACCTGC

UL42-R TTTCCCCGTACACCGTCTTG

UL52-F CGTCAAACACAACGTGACCC

UL52-R GCCAAACGCCCCATCATTTT

GAPDH-F CAAGAAGGTGGTGAAGCAGGC

GAPDH-R CATACCAGGAAATGAGCTTGAC
